# Supplementary figures and images for: Luteolin Improves Perivascular Adipose Tissue Profile and Vascular Dysfunction in Goto-Kakizaki Rats
Source: Int J Mol Sci. 2021 Dec 20;22(24):13671. doi: 10.3390/ijms222413671 (PMC8706309; doi:10.3390/ijms222413671)

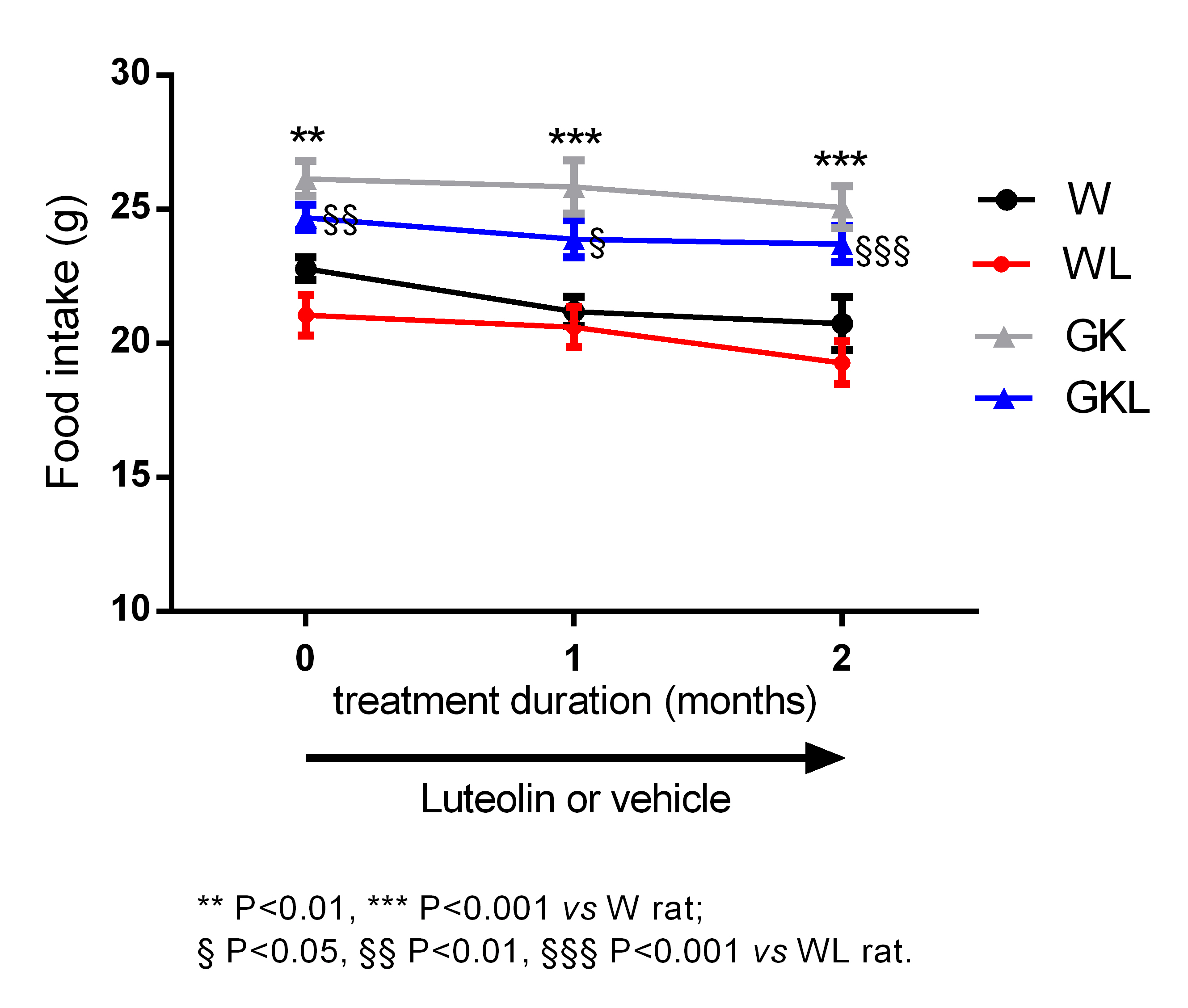

Supplement: Supplementary file 1 [file ijms-22-13671-s001.zip › ijms-1477633-supplementary.jpg]

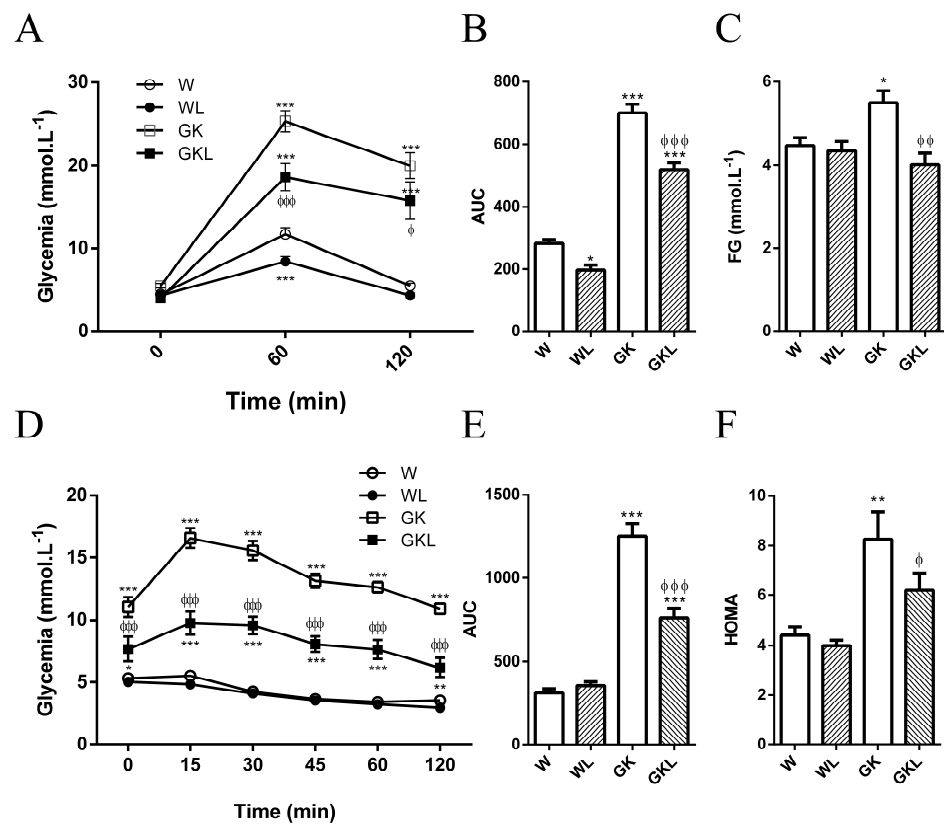

Figure S1

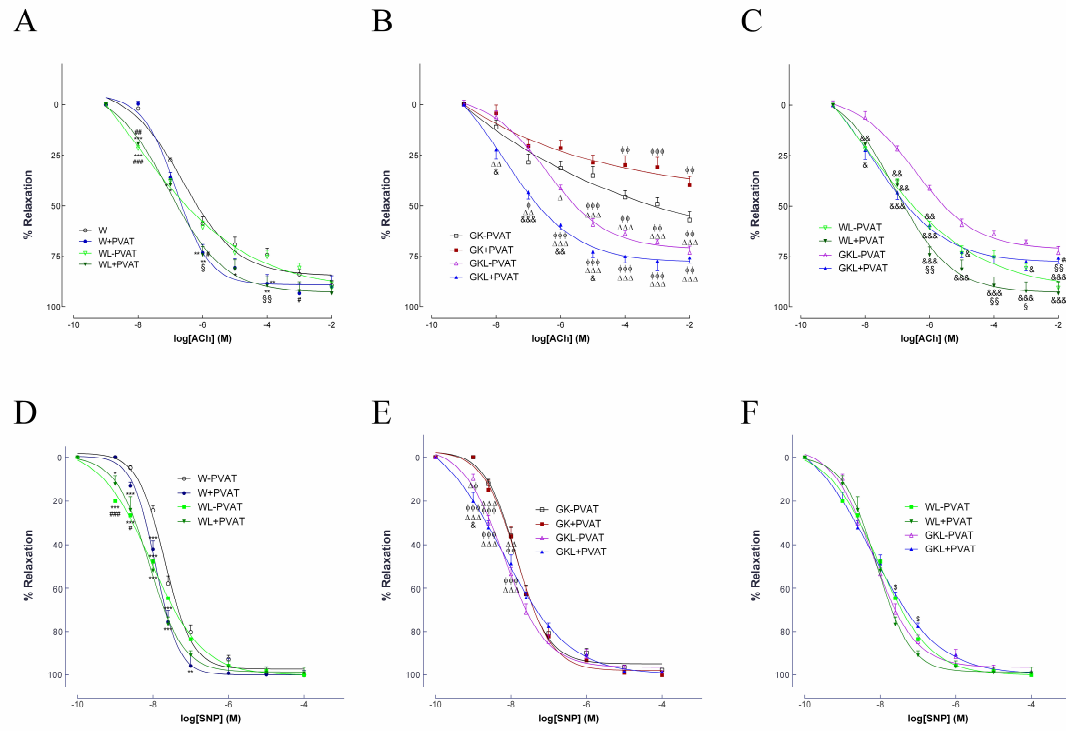

Figure S2

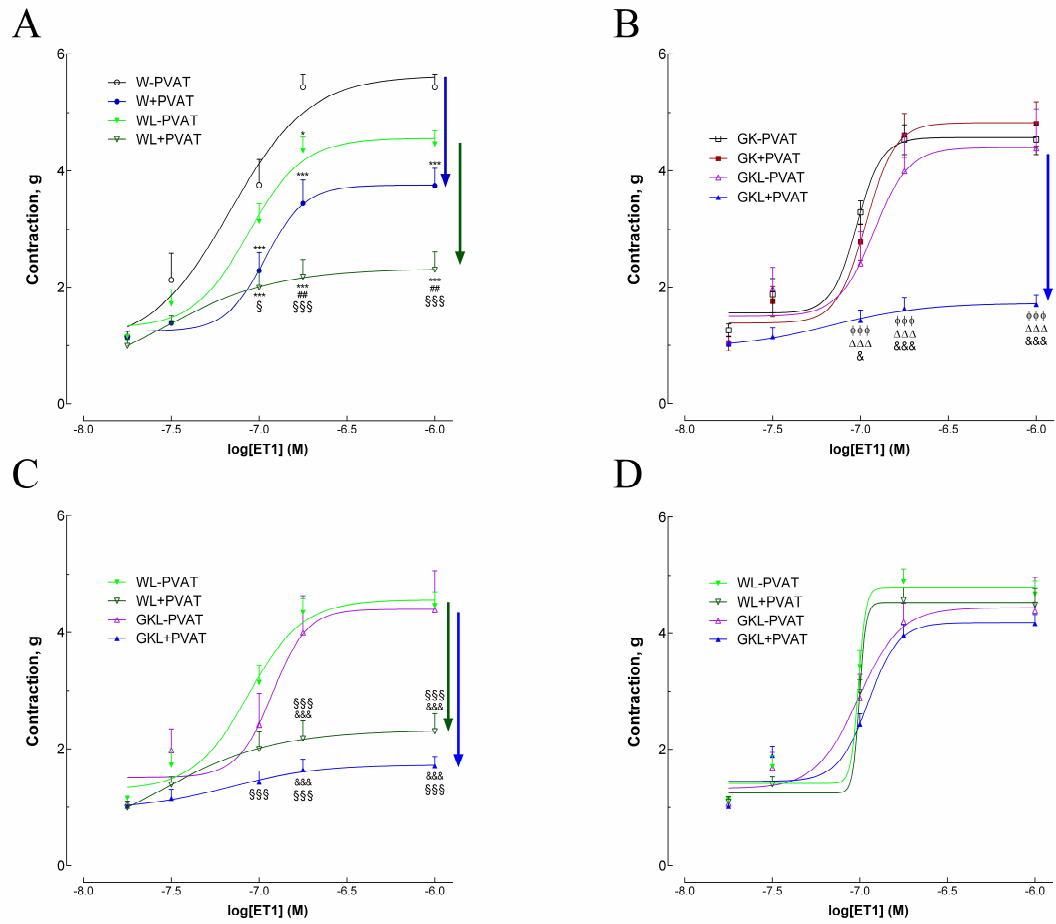

Figure S3

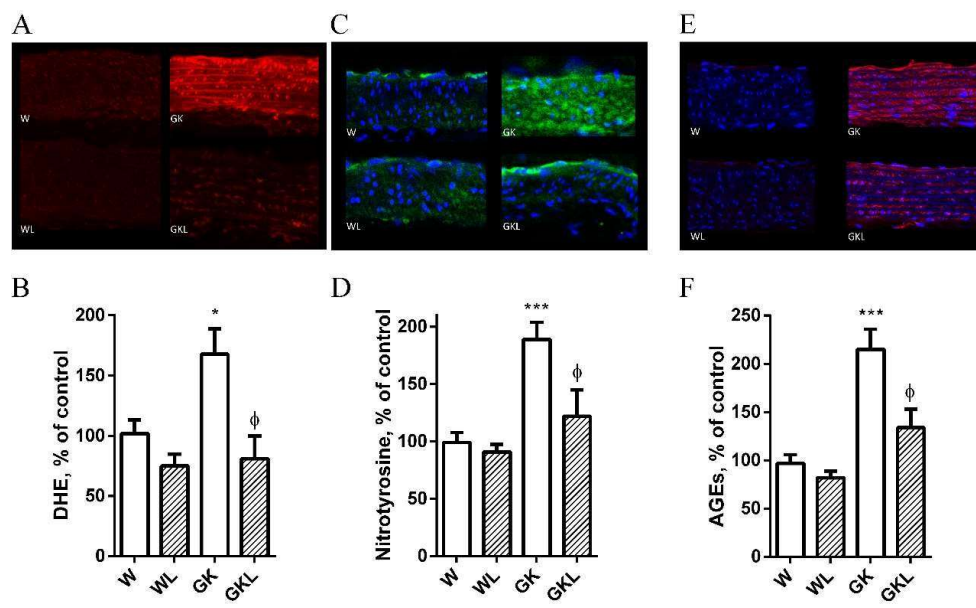

Figure S4

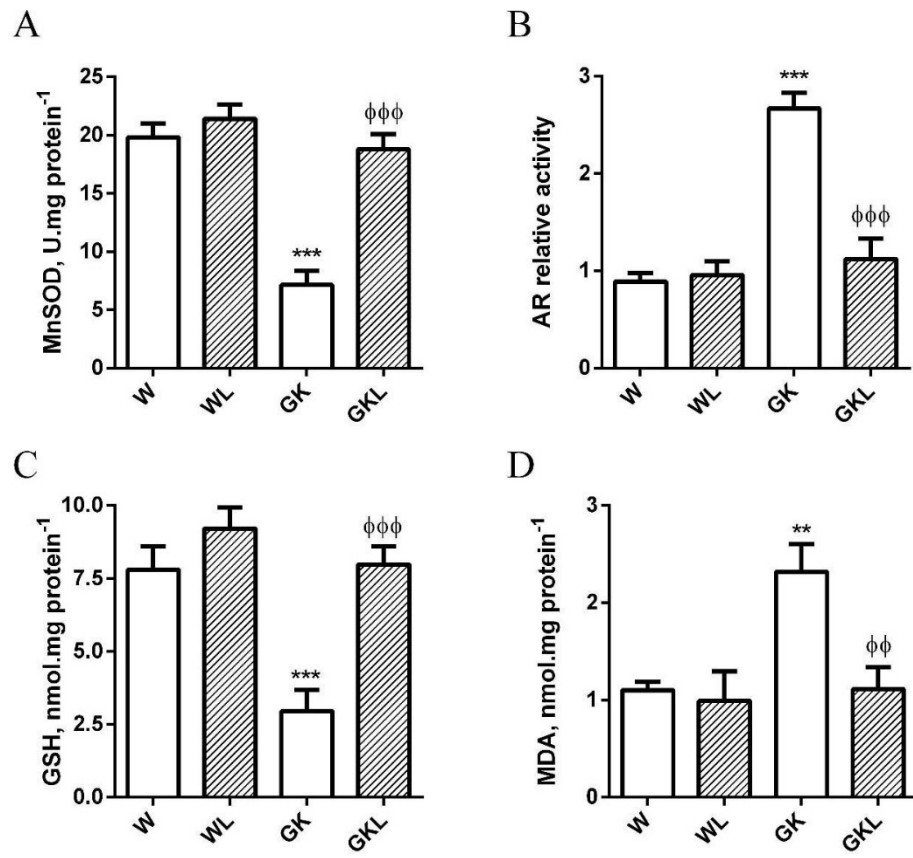

Figure S5

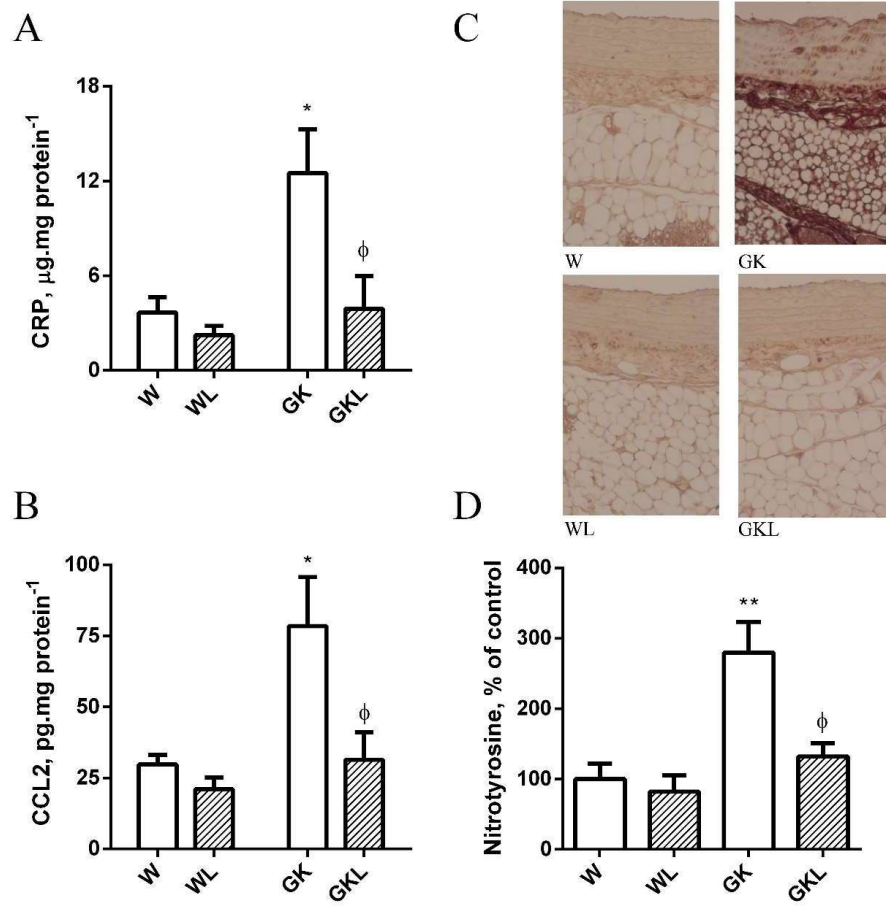

Figure S6

Supplement: Supplementary file 1 [file ijms-22-13671-s001.zip › ijms-1477633_figures.pdf]
